# Efficient Electrochemical CO_2_ Reduction Using AgN_3_ Single‐Atom Sites Embedded in Free‐Standing Electrodes for Flow Cell Applications

**DOI:** 10.1002/smsc.202400643

**Published:** 2025-07-06

**Authors:** M. Nur Hossain, Ali Malek, Zhangsen Chen, Lei Zhang, Shuhui Sun, Hanshuo Liu, Roberto Neagu, Jigang Zhou, Hui Yuan, Christopher S. Allen, Gianluigi Botton

**Affiliations:** ^1^ Clean Energy Innovation Research Center National Research Council of Canada Vancouver BC V6T 1W5 Canada; ^2^ Centre Énergie Matériaux Télécommunications Institut National de la Recherche Scientifique (INRS) Varennes Québec J3X 1P7 Canada; ^3^ Canadian Light Source Saskatoon Saskatchewan S7N 2V3 Canada; ^4^ Department of Materials Science and Engineering McMaster University Hamilton Ontario L8S 4L7 Canada; ^5^ Department of Materials University of Oxford Parks Road Oxford OX1 3PH UK; ^6^ Electron Physical Science Imaging Center Diamond Light Source Ltd. Harwell Science and Innovation Campus Chilton Didcot OX11 0DE UK

**Keywords:** CO_2_ reduction, electrolysis, free‐standing electrodes, silver, single‐atom catalysts

## Abstract

The electrochemical reduction of CO_2_ into valuable chemicals presents a promising strategy for carbon utilization; however, it remains challenging due to low activity, poor selectivity and stability of existing catalysts. In this study, we report the fabrication of free‐standing silver single‐atom catalysts (Ag SACs) designed for the efficient conversion of CO_2_ to carbon monoxide (CO) at high current densities in a bicarbonate electrolyzer. The Ag single atoms dispersed within a carbon matrix, forming Ag—N_3_ active sites for the electrocatalytic CO_2_ reduction reaction (CO_2_ RR). The catalytic activity and stability of the free‐standing Ag SACs are evaluated at a current density of 100 mA cm^−2^, demonstrating prolonged electrolysis with consistent Faradaic efficiency for CO production. Density functional theory calculations revealed that the Ag—N_3_ active site significantly lowers the energy barriers for the CO_2_ absorption step compared to Ag—Ag and Ag—Ni sites, facilitating CO_2_ activation and contributing to enhanced catalytic activity and stability during CO_2_ reduction. Detailed analysis of the electronic structure and coordination environment further validates the superior performance of the Ag—N_3_ site in the free‐standing Ag SACs, underscoring their effectiveness in CO_2_ electroreduction. These findings highlight the potential of the free‐standing Ag SACs to advance CO_2_ reduction technologies, offering enhanced efficiency and selectivity for CO_2_ conversion.

## Graphical abstract

This graphical abstract illustrates the performance of a free‐standing Ag single‐atom catalyst (Ag SAC) electrode designed for CO_2_ reduction in a bicarbonate electrolyzer. Compared to Ag nanoparticles and Ag SAC‐free electrodes, the Ag SAC electrode exhibits superior activity and significantly higher CO production with excellent Faradaic efficiency at higher current densities and lower electrode potentials, highlighting its effectiveness in CO_2_ conversion.

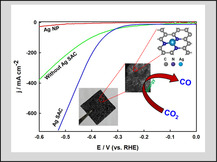

## 1 Introduction

**Figure 1:**
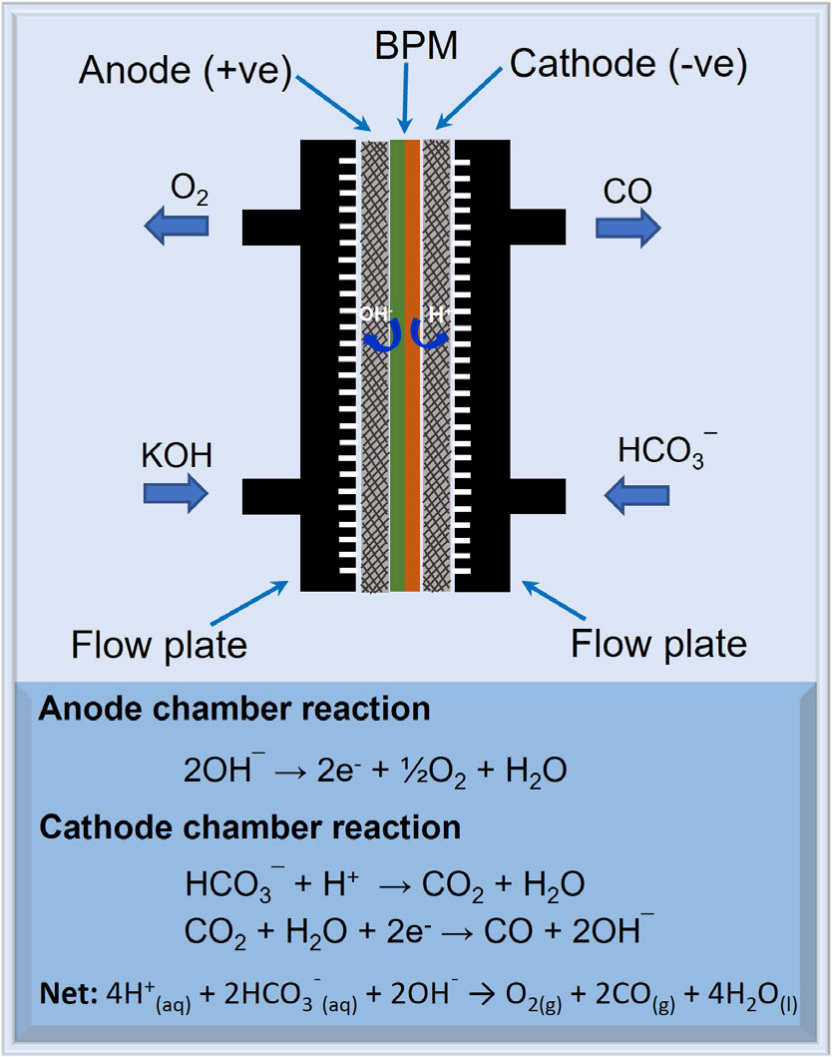
Schematic representation of a MEA for a bicarbonate electrolyzer, comprising flow plates, an anode, a BPM, and a cathode. Oxygen is produced at the anode through the OER, while the BPM facilitates water dissociation. At the cathode, sequential reactions occur: bicarbonate combines with protons to generate CO_2_ in situ, which is then electrochemically converted into CO and OH^−^.

The rising concentration of atmospheric CO_2_ is a primary driver of climate change, necessitating effective carbon utilization strategies.^[^ ^1^, ^2^ ^]^ Among these, the electrochemical CO_2_ reduction reaction (CO_2_ RR) has gained significant attention as a promising method to convert CO_2_ into valuable chemicals, such as carbon monoxide (CO), particularly when powered by renewable electricity.^[^ ^3^, ^4^ ^]^ The electrochemical reduction of CO_2_ requires high overpotentials because CO_2_ functions as an inert molecule, which leads to slow reaction kinetics and low selectivity. The complex multielectron transfer steps and the competitive hydrogen evolution reaction cause these problems when operating in aqueous electrolytes.^[^ ^5^, ^6^, ^7^ ^]^ Developing efficient, stable, and selective electrocatalysts is a crucial requirement to overcome these limitations. Furthermore, efficient CO_2_ delivery to the electrode surface is essential for improving reduction rates and product selectivity.^[^ ^8^, ^9^ ^]^ While gaseous CO_2_ electrolyzers show strong performance at the gas diffusion electrode (GDE) system, their industrial use is limited by the need for high‐purity CO_2_, which requires costly purification.^[^ ^10^, ^11^ ^]^ Bicarbonate electrolyzers offer a practical alternative by directly reducing HCO_3_^−^ from capture solutions, avoiding CO_2_ regeneration and gas handling. When paired with bipolar membranes (BPMs), they create an acidic environment at the cathode, where protons react with HCO_3_^−^ to form CO_2_, which is then reduced to CO on the GDE (**Figure** **1**).^[^ ^12^, ^13^, ^14^ ^]^ This setup boosts local CO_2_ concentration, improves efficiency without excess CO_2_ input,^[^ ^15^, ^16^, ^17^ ^]^ and regenerates OH^−^ to sustain continuous CO_2_ capture.

While membrane, electrolyte, and membrane electrode assembly (MEA) design affect bicarbonate electrolyzer performance, this work focuses on catalyst development to improve CO_2_ reduction efficiency. Nanostructured catalysts show enhanced activity in electrochemical CO_2_ reduction due to their tunable size, shape, and surface properties, with performance largely governed by the accessibility and nature of active sites. When reduced to the atomic scale, each metal atom can function as an active site, significantly boosting activity and selectivity.^[^ ^18^, ^19^, ^20^, ^21^ ^]^ Although many catalytic reactions require multi‐atom sites for molecular activation,^[^ ^22^, ^23^ ^]^ single‐atom catalysts (SACs) overcome this limitation by offering nearly 100% atom utilization and improved cost‐efficiency. Metals such as Fe, Mn, Ni, Co, Cu, Zn, Ag, Pd, and Sn have been widely studied in SAC form.^[^ ^24^, ^25^, ^26^, ^27^ ^]^ SACs provide well‐defined, uniform active sites, enabling controlled selectivity and bridging the gap between heterogeneous and homogeneous catalysis.^[^ ^28^, ^29^, ^30^ ^]^ Nitrogen‐coordinated SACs, in particular, are promising due to the stable M–N–C configuration. Strategies like heteroatom doping and defect engineering on carbon supports have proven effective for stabilizing single atoms and tuning their electronic environments.^[^ ^31^, ^32^ ^]^ Despite progress in achieving high metal loadings and precise atomic dispersion, long‐term stabilization of single atoms remains a major challenge.^[^ ^33^, ^34^ ^]^

Among noble metals, silver (Ag) has emerged as a cost‐effective and active catalyst for CO_2_‐to‐CO conversion, a key step in downstream fuel and chemical production.^[^ ^35^, ^36^ ^]^ In SAC form, Ag coordinated with nonmetals (e.g., N, O, S) forms unique local environments. Ag–N_x_ structures, where Ag carries a partial positive charge due to nitrogen's electronegativity, enhance the binding of intermediates like *COOH, improving reaction rates.^[^ ^37^, ^38^ ^]^ Studies on Ag_1_/MnO_2_ SACs have demonstrated superior activity and selectivity compared to conventional Ag catalysts, though further tuning is needed to overcome rate‐limiting steps identified via density functional theory (DFT) analysis.^[^ ^39^, ^40^ ^]^ Traditional powdered catalysts are typically cast onto supports using binders like Nafion or polyvinylidene difluoride, which can block active sites, limit pore accessibility, and reduce the electrical conductivity.^[^ ^41^, ^42^ ^]^ Free‐standing SAC electrodes (FSSACs) address these limitations by eliminating the need for binders while maintaining structural integrity. These electrodes are fabricated using methods such as nanostructure deposition, hierarchical assembly, and chemical grafting. Notable FSSACs include Cu, Mn, Fe, Sn, Ni, and Co SACs embedded in supports like carbon membranes, carbon paper, and doped polyanilines.^[^ ^43^, ^44^, ^45^ ^]^ Such architectures improve electron transport, catalyst accessibility, and mechanical stability, enabling high‐performance CO_2_ reduction under practical conditions.

In this study, we developed free‐standing Ag SACs for efficient electrochemical reduction of CO_2_ using a bicarbonate electrolyzer, which showed excellent electrochemical performance and high Faradaic efficiency (FE) for CO production. We employed a novel and facile in situ chemical vapor deposition (CVD) method to synthesize these Ag SACs. The synthesis process started with 2‐methylimidazole depositing onto Ag‐doped ZnO nanoparticles, followed by pyrolysis at high temperature to produce well‐dispersed Ag single sites within porous carbon supported by Ni foam. The extended X‐ray absorption fine structure (EXAFS) was used to determine Ag coordination environment, while high‐angle annular dark‐field scanning transmission electron microscopy (HAADF‐STEM) was employed to observe isolated Ag single atoms. The synthesized free‐standing Ag SACs, particularly with Ag coordinated to nitrogen (Ag—N_3_), exhibited a low onset potential for CO_2_ RR (−0.27 V), a high maximum FECO of 60.2% at −0.35 V, and a CO partial current density (J_CO_) of 60 mA cm^−2^. The catalysts also showed remarkable stability, maintaining a steady‐state potential over 20 h at 100 mA cm^−2^ current density, surpassing the performance of previously reported Ag‐based catalysts. DFT analysis identified pyridinic Ag—N_3_ moieties as the primary active sites responsible for CO_2_ reduction to CO. DFT further revealed that the Ag—N_3_ site lowers the energy barrier for CO_2_ adsorption by stabilizing and activating the molecule through concurrent interactions between CO_2_ atoms and the Ag centers.

## 2 Results and Discussion

**Figure 2:**
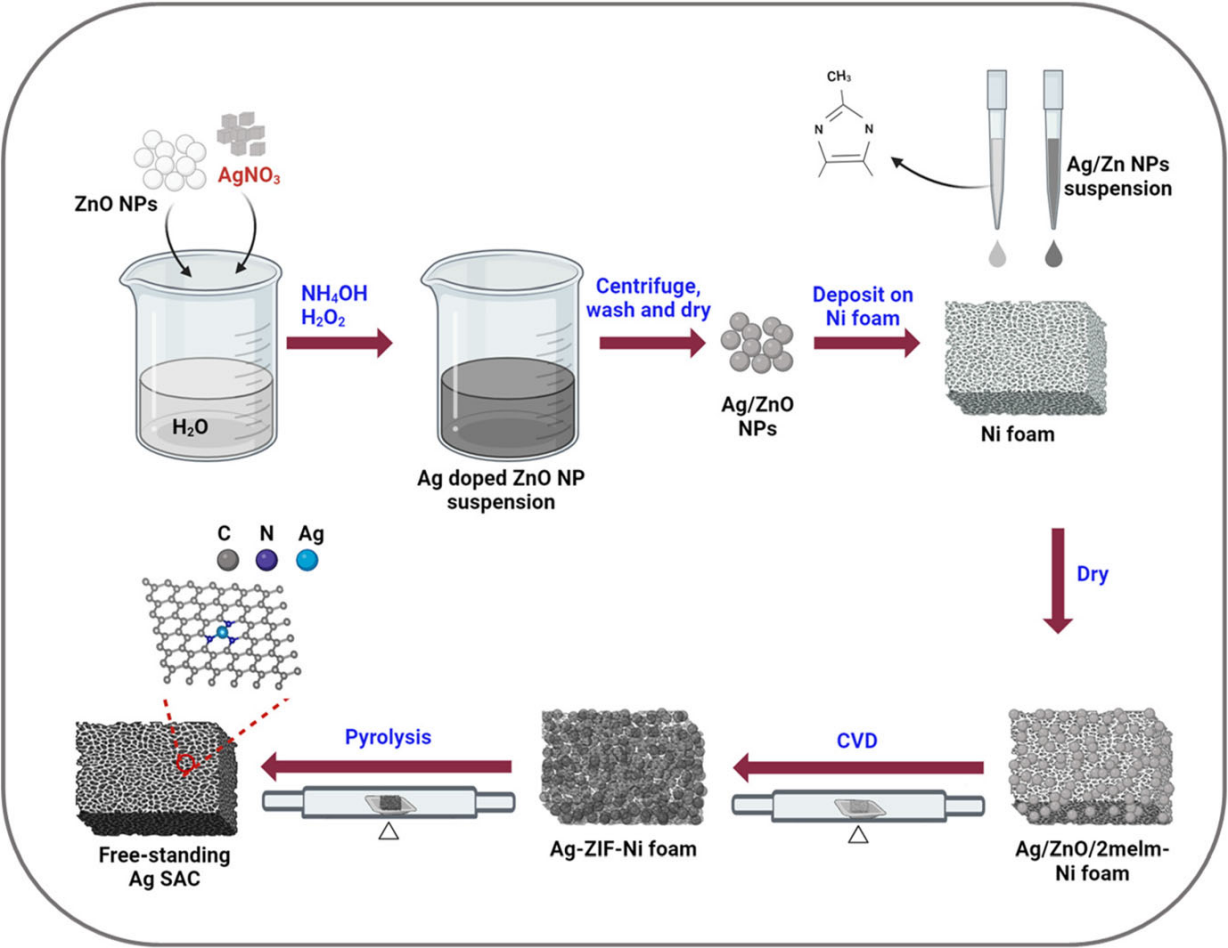
Schematic depiction of the synthesis process for the free‐standing Ag SAC electrode.

**Figure** **2** illustrates the synthetic scheme for the free‐standing Ag‐SACs. The synthesis process began with mixing ZnO nanoparticles and AgNO_3_ in water, followed by the addition of NH_4_OH and H_2_O_2_ to deposit Ag particles onto the ZnO nanoparticles. Next, a 400 mg mL^−1^ solution of 2melm was cast onto a Ni foam, and the Ag–ZnO nanoparticles were subsequently deposited onto the formed 2melm/Ni foam. The resulting 2melm–Ag–ZnO/Ni foam was subjected to in situ CVD of 2melm on the ZnO–Ag particles at 300 °C, forming ZIF–8@Ag particles. The X‐ray diffraction (XRD) patterns of Ag–ZnO and Ag/ZnO–ZIF are presented in Figure S2, Supporting Information. The analysis shows strong peaks at 2*θ* = 7.35, 10.37, 12.75, 14.75, 16.48, and 18.05°, corresponding to the (110), (200), (211), (220), (310), and (222) planes, respectively, indicating high crystallinity. The observed XRD patterns closely match the simulated pattern listed in JCPDS 00‐062‐1030, confirming the structural integrity of the synthesized ZIF‐8.^[^ ^46^, ^47^, ^48^, ^49^ ^]^ Finally, pyrolysis at 900 °C was carried out, yielding the free‐standing Ag–N–C electrode. The synthesized free‐standing Ag–N–C electrode, showcased in Figure S3, Supporting Information, is robust and flexible, retaining its initial structure even under bending stress. Moreover, the electrode maintains its original form after a 20 h stability test, indicating strong mechanical stability.

**Figure 3:**
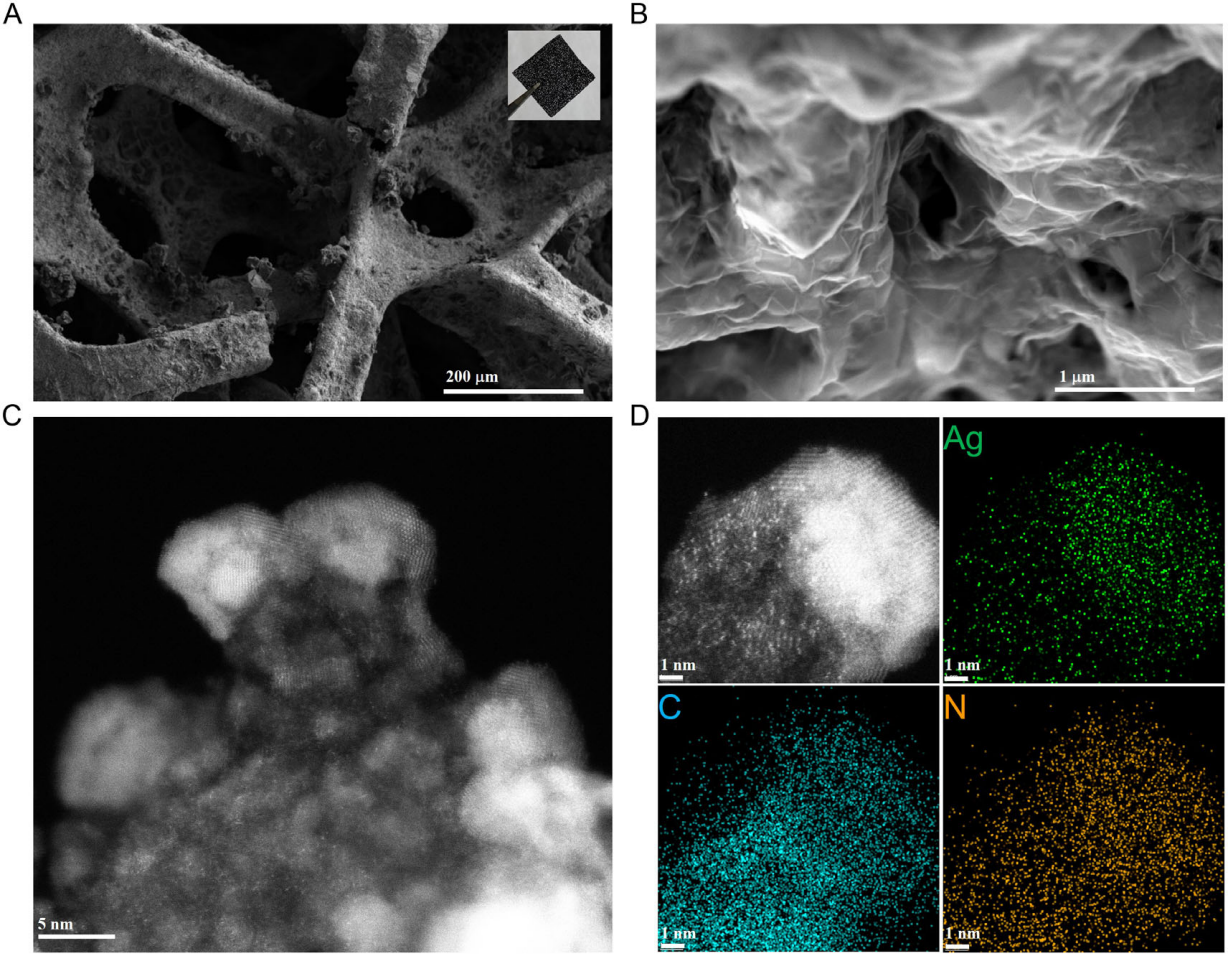
Characterization of the free‐standing Ag SAC electrode: A) SEM image of the Ag SAC electrode structure (inset shows the completely fabricated electrode). B) High‐magnification SEM image of the Ag SAC electrode surface. C) Aberration‐corrected HAADF‐STEM image of the Ag SAC electrode. D) Elemental mapping illustrating the distribution of silver (Ag) in green, carbon (C) in cyan, and nitrogen (N) in yellow.

The scanning electron microscope (SEM) technique was employed to analyze the morphologies of plain nickel foam and Ag SACs grown on Ni foam. Initial SEM images of plain nickel foam are provided in Figure S4, Supporting Information. This nickel substrate exhibits a cross‐linked structure with pore sizes ranging from 200 to 500 μm, as shown in Figure S4A, Supporting Information. High‐resolution SEM (HR‐SEM) images of the Ni foam (Figure S4B, Supporting Information) reveal varying surface roughness. After the growth of Ag SACs on Ni foam through CVD and pyrolysis steps, SEM analysis reveals a dense and uniform coverage of the nickel foam's porous network by a composite, with Ag SACs distributed throughout the carbon matrix, as shown in **Figure** **3**A. The formed Ag SAC on Ni foam is shown in the inset of Figure 3A. The HR‐SEM image further illustrates the formation of a film over the Ni foam surface, with no visible Ag nanoparticles, following Ag SAC deposition, as shown in Figure 3B. Aberration‐corrected HAADF‐STEM analysis confirmed the distribution of Ag atoms throughout the carbon matrix. As shown in Figure 3C, bright dots uniformly dispersed across the Ni surface represent the Ag atoms integrated into the carbon matrix. The Ag atoms and Ni lattice fringes are clearly distinguishable due to the sensitive Z‐contrast of the heavier elements, illustrating the Ag SAC structure of the materials. Notably, no visible Ag nanoparticles or nanoclusters are present on the Ni surface throughout the entire HAADF‐STEM image region, which is consistent with the SEM analysis. Additionally, the elemental mapping images in Figure 3D display a uniform distribution of C, N, and Ag throughout the carbon matrix on the Ni surface.

**Figure 4:**
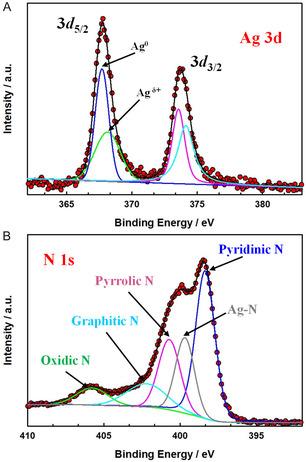
High‐resolution XPS spectra of the free‐standing Ag SAC electrode. A) Ag 3d and B) N 1s.

**Figure 5:**
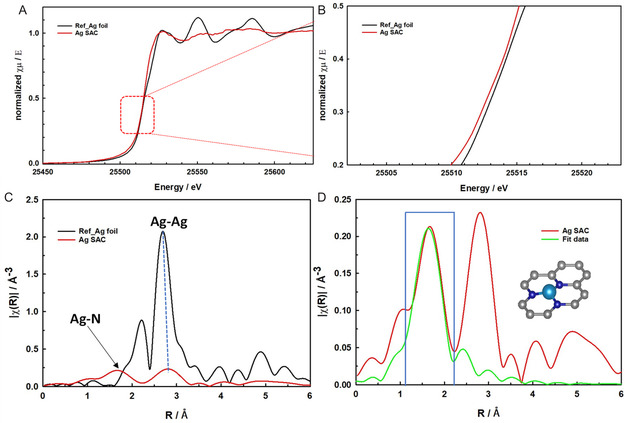
Electronic states of the Ag atom in the free‐standing Ag SAC electrode: A) Ag K‐edge XANES spectra comparing the Ag SAC and reference Ag foil. B) Enlarged view of the rising edge. C) Fourier‐transformed (FT) k^2^‐weighted EXAFS spectra of the Ag SAC and Ag foil. D) Corresponding EXAFS R‐space fitting curves for the Ag SAC with a schematic model inset illustrating the Ag SAC structure, where Ag (light blue) is bonded with three pyridinic nitrogen atoms (N in blue); carbon atoms are shown in gray.

**Figure 6:**
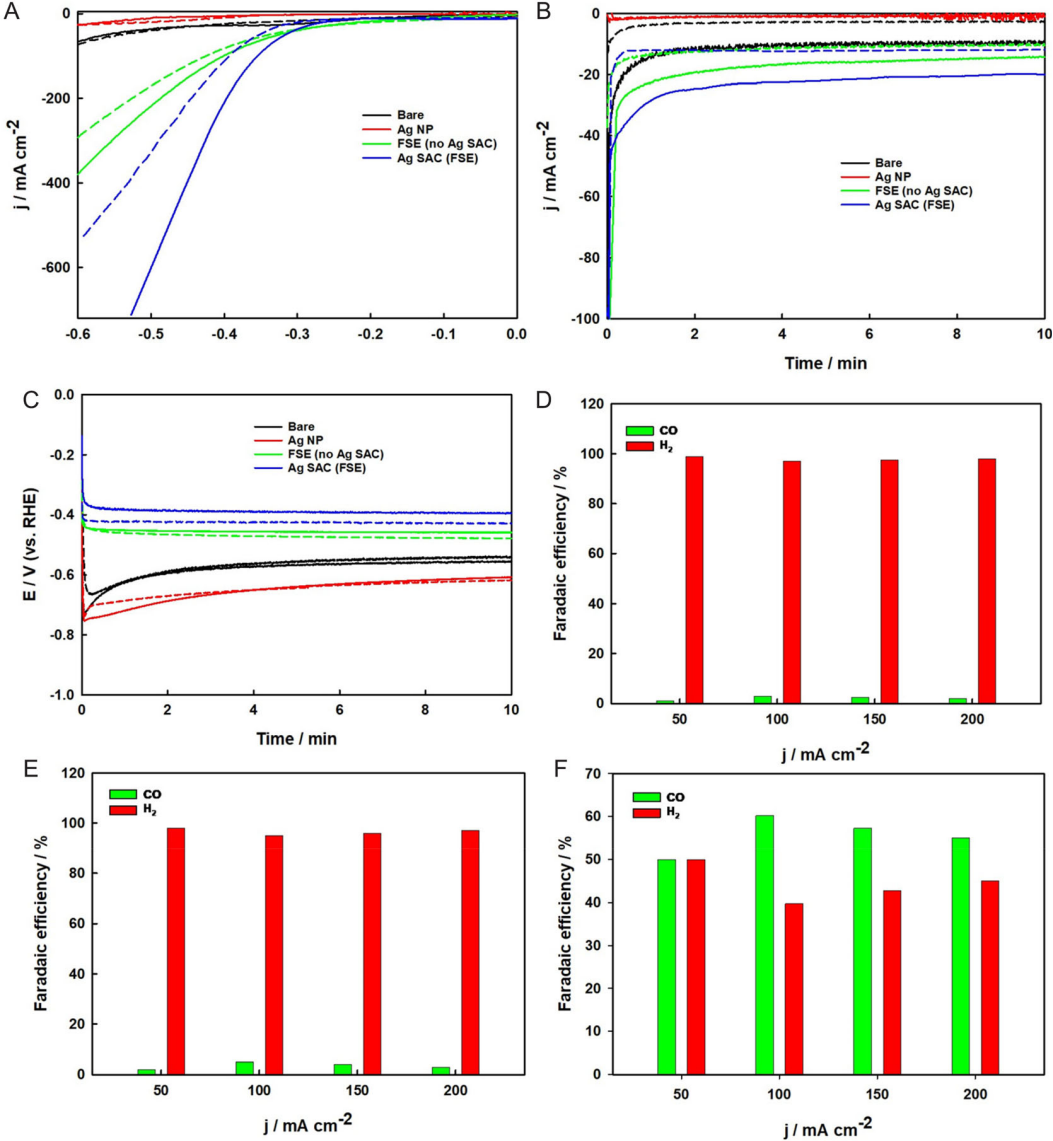
Electrocatalytic performances of various electrodes in the CO_2_ RR: A) LSV curves obtained at a scan rate of 20 mV s^−1^, B) CA curves recorded at −0.35 V (vs RHE), and C) CP curves measured at −100 mA cm^−2^. Broken lines represent Ar‐saturated electrolytes, and solid lines represent CO_2_‐saturated electrolytes. FE for CO and H_2_ at various applied current densities are shown for D) bare Ni foam, E) free‐standing Ni/C foam without the Ag SAC, and F) free‐standing Ag SAC electrode.

X‐ray photoelectron spectroscopy (XPS) analysis was conducted to investigate the surface chemical state of the sample information, focusing on key elements such as carbon (C 1s), Silver (Ag 3d), Nitrogen (N 1s), and Oxygen (O 1s), as illustrated in Figure S5, Supporting Information. Notably, the Ag 3d core levels exhibit a shift to lower energy, suggesting the existence of multiple oxidation states of silver, as shown in **Figure** **4**A. The deconvolution of the Ag 3d spectrum reveals two peaks: one at 367.7 eV, which is assigned to metallic silver (Ag^0^), potentially indicating an Ag—Ni bond formation due to the use of Ni as a substrate, and another at 368.1 eV, corresponding to oxidized Ag species.^[^ ^50^, ^51^, ^52^, ^53^ ^]^ This suggests an oxidation state for Ag between 0 and +1, consistent with values reported in the literature. The atomic percentage of silver is estimated to be around 1%. The overall atomic percentage of nitrogen is ≈11%. High‐resolution N 1s XPS spectra, presented in Figure 4B, show a shift of the N 1s signal to a higher binding energy after the introduction of Ag, which supports the formation of Ag—N bonds and indicates strong interaction and electron transfer between the nitrogen and Ag species. The N1s spectrum is deconvoluted into five distinct nitrogen species: pyridinic N at 398.4 eV, pyrrolic N at 400.7 eV, graphitic N at 402.2 eV, oxidic N at 405.9 eV, and Ag—N at 399.6 eV.^[^ ^54^, ^55^ ^]^ Quantification of these species reveals that pyridinic N constitutes 40% of the nitrogen content, pyrrolic N 20%, graphitic N 12.45%, oxidic N 7.2%, and Ag—N 19.2%. The significant proportion of Ag—N (19.2%) highlights the presence of Ag—N3 single‐atom sites in the developed Ag SAC. To further investigate the electronic structure and coordination environment of the Ag SAC, X‐ray absorption near‐edge spectra (XANES), and EXAFS measurements were conducted. The normalized Ag K‐edge absorption spectra of both the Ag foil and Ag SAC are presented in **Figure** **5**A. The edge position in the Ag SAC (red line) confirms its metallic nature.^[^ ^40^, ^56^, ^57^ ^]^ The absence of resonance peaks at 25 550 eV and 25 558 eV in the Ag SAC spectrum suggests an amorphous structure, indicating that the single Ag atoms exhibit characteristics akin to an amorphous state.^[^ ^52^, ^55^, ^58^ ^]^ Figure 5B demonstrates a slight shift of the absorption edge to lower energy positions compared to the Ag foil, implying that the Ag atoms in the SAC are slightly negatively charged (denoted as Ag^*δ−*^, where *δ* represents an uncertain value). This shift may be directly related to strong metal‐support interactions. This negative shift is similar to what is observed in Au_1_Ag_1_ alloys and could be attributed to charge transfer from the nickel substrate used to grow the Ag SAC.^[^ ^59^ ^]^ The coordinating environments of Ag were investigated using EXAFS, as presented in Figure 5C. The Fourier transform of the k^2−^ weighted χ(k) function from the EXAFS spectra of the Ag SAC revealed a primary peak at ≈1.67 Å, corresponding to the first shell of Ag—N coordination.^[^ ^51^, ^55^ ^]^ An additional significant peak at 2.8 Å, slightly higher than the typical Ag—Ag bond (≈2.7 Å) observed in Ag foil, indicates bond distortion or an altered coordination environment in the metal–metal bonds. This deviation may result from interactions between the Ag atoms and the Ni support, potentially forming an Ag—Ni coordination bond. Consequently, the synthesized Ag SAC may consist of both single‐atom Ag and Ag—Ni coordination bonds. The quantitative coordination configuration of the Ag atom in the Ag SAC was determined via EXAFS fitting, focusing on the 1.15–2.2 Å range for the Ag—N path, as depicted in **Figure** **6**D. According to the fitting data, Ag in the Ag SAC is coordinated with three nitrogen atoms, presenting a coordination number of 2.998 and a bond distance of 1.634 Å. This arrangement suggests a strong interaction between the Ag atoms and the surrounding carbon matrix, making AgN3 the primary reactive site. Due to the low Ag metal content, the K‐space spectra for the Ag SAC, shown in Figure S6, Supporting Information, exhibit poor quality with high noise levels in the higher K‐region. Therefore, the R‐space results discussed earlier were derived from the K‐region between 2 and 9 Å, where the signal quality remains sufficient.

The electrocatalytic performance of the synthesized free‐standing Ag SAC electrodes was evaluated using linear sweep voltammetry (LSV), chronoamperometry (CA), and chronopotentiometry (CP) techniques in both Ar and CO_2_‐saturated 2.0 M KHCO_3_ solutions, with pH values of 8.4 and 7.8, respectively. Figure 6A showcases the LSV curves for bare Ni foam, Ag nanoparticles, free‐standing Ni/C foam without the Ag SACs, and free‐standing Ag SAC electrodes, all recorded at a scan rate of 20 mV s^−1^. The current density in CO_2_‐saturated KHCO_3_ was significantly higher than in Ar‐saturated KHCO_3_, illustrating the electrodes’ catalytic activity for CO_2_ reduction. Notably, the free‐standing Ag SAC electrode exhibited higher current densities and a lower onset potential compared to the other electrodes in CO_2_‐saturated KHCO_3_. For example, at −0.5 V, the cathodic current for the free‐standing Ag SAC reached 610.51 mA cm^−2^, far surpassing those of free‐standing Ni/C foam without the Ag SACs (215.25 mA cm^−2^), Ag nanoparticles (12.37 mA cm^−2^), and bare Ni foam (36.10 mA cm^−2^), and outperforming most previously reported Ag‐based SAC catalysts.^[^ ^40^, ^51^, ^52^, ^55^, ^60^, ^61^, ^62^ ^]^ This trend was consistent in the steady‐state current densities measured at −0.35 V, as shown in the CA curves (Figure 6B), where the free‐standing Ag SAC outperformed all other electrodes. Similarly, the steady‐state potentials measured at −100 mA cm^−2^, shown in the CP curves (Figure 6C), confirmed that the free‐standing Ag SAC achieved the lowest electrode potential. Across all LSV, CA, and CP assessments, the free‐standing Ag SAC demonstrated superior electrocatalytic activity compared to all other electrodes fabricated in this study, indicating its potential as an effective electrocatalyst for CO_2_ reduction. Additionally, the support effect was analyzed by comparing the CO_2_ reduction activity of the free‐standing Ag SAC on Ni and carbon supports, as depicted in Figure S7, Supporting Information. The Ag SAC on Ni foam showed a higher current density in the LSV (Figure S7A, Supporting Information), greater current density at −0.35 V in the CA (Figure S7B, Supporting Information), and a lower electrode potential at −100 mA cm^−2^ in the CP (Figure S7C, Supporting Information), suggesting a stronger interaction with the Ni support compared to the carbon support.

Electrochemical impedance spectroscopy (EIS) was used to assess the charge‐transfer resistance associated with CO_2_ reduction. Nyquist plots for bare Ni Foam, Ag NPs, Ni/C foam without the Ag SAC, and the free‐standing Ag SAC electrodes were obtained in CO_2_‐saturated 2.0 M KHCO_3_ solutions at −0.35 V (vs. reversible hydrogen electrode (RHE)), as shown in Figure S8A, Supporting Information. The impedance spectra for all electrodes featured a semicircle in the low‐frequency range, indicative of charge‐transfer resistance.^[^ ^63^, ^64^ ^]^ An equivalent electrical circuit, shown in the inset, accurately fitted the experimental data using Z‐view software, with results and errors summarized in Table S1, Supporting Information. Solution resistances (Rs) were low, and constant phase element values above 0.7 indicated capacitive behavior. The free‐standing Ag SAC electrode exhibited substantially lower charge‐transfer resistance (24.72 Ω cm^2^), compared to the other tested electrodes, highlighting its superior catalytic activity for CO_2_ reduction. Further EIS measurements showed a reduction in resistance from 24.72 Ω cm^2^ to 4.12 Ω cm^2^ as the cathodic potential increased from −0.35 V to −0.45 V (Figure S8B, Supporting Information), affirming the electrode's increased electrocatalytic efficiency. To further explore the kinetics of CO_2_ reduction, Tafel plots were measured for bare Ni foam, Ag NPs, Ni/C foam without the Ag SAC, and the free‐standing Ag SAC electrodes using LSV in CO_2_‐saturated 2.0 M KHCO_3_ electrolyte. The Tafel slopes were measured as 105 mV dec^−1^ for bare Ni Foam, 121 mV dec^−1^ for Ag NPs, 93 mV dec^−1^ for Ni/C foam without Ag SAC, and 72 mV dec^−1^ for the free‐standing Ag SAC electrodes, as shown in Figure S8C, Supporting Information. The significantly lower Tafel slope of the free‐standing Ag SAC electrodes demonstrates a faster initial electron transfer in CO_2_ reduction to CO compared to the other electrodes tested.^[^ ^40^, ^65^ ^]^

To assess the selectivity and efficiency of these catalysts in the CO_2_ RR, bulk electrolysis was conducted at various current densities for 2 h. During this time, CO_2_ was continuously supplied to the solution, and the electrolyte was circulated between the CO_2_ absorber column and the electrochemical cell. Figures 6D, 6E, and 6F display the FE for bare Ni foam, Ni/C foam without the Ag SAC, and free‐standing Ag SAC electrodes at current densities of 50, 100, 150, and 200 mA cm^−2^, respectively. Both the bare Ni foam and the Ni/C foam without the Ag SAC primarily produced hydrogen under all tested conditions, with the FE for CO below 5% across all current densities. In contrast, the free‐standing Ag SAC electrode demonstrated strong selectivity for CO production, achieving FEs of ≈50%, 60%, 57.3%, and 55% at current densities of 50, 100, 150, and 200 mA cm^−2^, respectively, as shown in Figure 6F. The remaining FE is attributed to hydrogen evolution, as confirmed by gas chromatograph (GC) analysis. This indicates that the presence of Ag single atoms effectively suppresses the hydrogen evolution reaction. The high FE for CO formation suggests that the active sites on the free‐standing Ag SAC electrode, particularly those involving Ag—N_3_ coordination, are highly effective for CO_2_ reduction.

**Figure 7:**
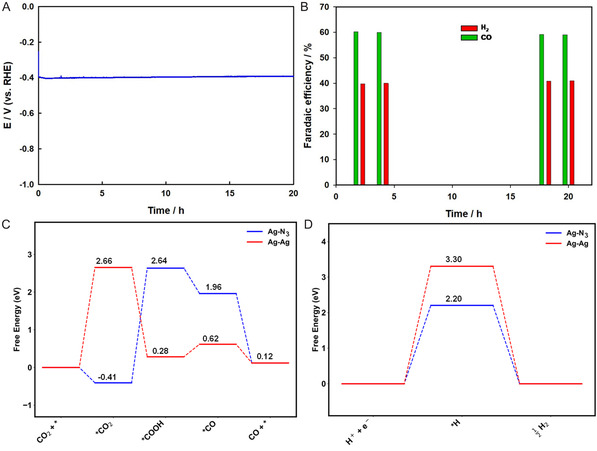
Stability test of the free‐standing Ag SAC electrode: A) CP recorded at −100 mA cm^−2^ in CO_2_‐saturated electrolyte over 20 h. B) Corresponding FE for CO and H_2_ measured during the first and last two hours. Free energy profiles for C) CO_2_ reduction and D) hydrogen evolution reaction with Ag—N_3_ and Ag—Ag.

The stability of the free‐standing Ag SAC electrode was evaluated at a constant current density of −100 mA cm^−2^ over 20 h using the chronoamperometric method, as illustrated in **Figure** **7**A. Impressively, the current density remained stable throughout the CO_2_ reduction process, highlighting the exceptional durability of the Ag SAC electrode. Aberration‐corrected HAADF‐STEM and XPS analyzes of the free‐standing Ag SAC electrode were performed after 20 h of electrochemical CO_2_ reduction, as presented in Figure S9, Supporting Information. The HAADF‐STEM image (Figure S9A, Supporting Information) reveals that the distribution of Ag atoms within the carbon matrix remains consistent with that observed prior to CO_2_ reduction (Figure 3C). Figure S9B, Supporting Information, shows elemental mapping images that confirm a uniform distribution of C, N, and Ag throughout the carbon matrix. Both the XPS survey spectra and high‐resolution Ag 3d XPS spectrum of the free‐standing Ag SAC electrode display the presence of Ag peaks that remain virtually unchanged before and after CO_2_ reduction, further validating the high stability of the electrode. Figure 7B shows the FE for CO production, which was maintained at 60.2% and 59% during the first and last two hours of the test, respectively. These results underscore the remarkable stability of the Ag SAC electrode for prolonged electrochemical CO_2_ reduction. The robust performance of the free‐standing Ag SAC electrode can be attributed to its large surface area and the uniform distribution of Ag—N_3_ active sites within the carbon matrix. The performance of several promising Ag‐based catalysts for electrochemical CO_2_ reduction, as recently reported in the literature, is compared in Table S2, Supporting Information. This comparison shows that the free‐standing Ag SAC developed in this study achieved much higher current efficiency at a relatively low overpotential and also exhibited superb stability. All the results presented confirm that the free‐standing Ag SAC developed in this study is a highly promising catalyst for the electrochemical reduction of CO_2_.

DFT calculations were employed to evaluate the catalytic activity of Ag—N_3_, Ag—Ag, Ag—Ni, and Ni sites in CO_2_ reduction and hydrogen evolution reactions. The analysis focused on the Gibbs free energies and optimized structures of key intermediates, including *COO, *COOH, *CO, and *H, which play critical roles in the reaction mechanisms.^[^ ^55^, ^66^, ^67^, ^68^, ^69^ ^]^ As depicted–in Figure 7C, S10A,S11, Supporting Information, the CO_2_ reduction process proceeds through four main stages. First, CO_2_ is adsorbed onto the catalyst surface (*), forming the *COO intermediate (CO_2_ + * → *COO). Next, a proton‐coupled electron transfer converts *COO to *COOH (*COO + H^+^ + e^−^ → *COOH). A subsequent transfer leads to the formation of *CO and H_2_O (*COOH + H^+^ + e^−^ → *CO + H_2_O). Finally, *CO desorbs as CO gas (*CO → CO + *), regenerating the catalyst for further reactions. Our DFT results reveal a mechanistic distinction between Ag—N_3_ and Ag—Ag: for Ag—N_3_, the rate‐determining step is CO2 adsorption, whereas for Ag—Ag, it is the formation of the *COOH intermediate. This difference plays a pivotal role in defining the catalytic behavior of each site. Notably, the *COO intermediate is significantly more stable on Ag—N_3_ compared to Ag—Ag and Ni. Ag—N_3_ exhibits the lowest energy barrier for *COO formation, at 0.41 eV, which is markedly lower than 2.65 eV for Ag—Ag and 0.71 eV for Ni. This favorable performance of Ag—N_3_ is attributed to its stronger adsorption characteristics for *COO on Ag—N_3_, as illustrated in Figure 7C and Figure S10A, Supporting Information. Furthermore, although *COOH forms in both Ag—N_3_ and Ag—Ag pathways, the adsorption energies vary considerably. As detailed in Tables S4, S5, Supporting Information, *COOH binds with an energy of –464.93 eV on Ag—N_3_ and –474.06 eV on Ag—Ag, a notable difference of over 9 eV, further confirming the distinct adsorption behavior at each active site. Additionally, while Ag—Ni demonstrates strong CO_2_ adsorption, it has a significantly higher energy barrier for CO formation (5.1 eV), compared to Ag—N_3_ (1.96 eV). This indicates that Ag—N_3_ enhances catalytic activity by significantly lowering the energy barriers for CO_2_ absorption and stabilizing CO_2_ intermediates more effectively than Ag—Ag and Ag—Ni, making it a superior catalyst for CO_2_ reduction. Further DFT studies on the binding energies of *COH and *HCO intermediates—key precursors for hydrocarbon production—revealed that their formation is less favorable compared to direct CO production. This suggests that Ag—N_3_ exhibits higher selectivity for CO over hydrocarbons (Figure S12, Supporting Information).^[^ ^70^, ^71^ ^]^ Hydrogen evolution reaction activity analysis further indicated that hydrogen evolution is predominantly feasible over Ag—Ni and Ni, as demonstrated by the potential‐specific free energy diagrams in Figure 7D and Figure S10B, Supporting Information. Overall, Ag—N_3_ demonstrated excellent performance, achieving a FE for CO of up to 60% at 100 mA cm^−2^ and 0.4 V.

## 3 Conclusion

In summary, we have developed a free‐standing Ag SAC electrode featuring Ag—N_3_ sites for the enhanced electrochemical reduction of CO_2_ to CO in a bicarbonate electrolyzer system. The synthesized electrode demonstrated excellent catalytic activity and stability, achieving a FE of 60.2% at −100 mA cm^−2^ over 20 h with only a 1.2% loss in activity. Comprehensive DFT calculations revealed that the Ag—N_3_ site significantly lowers the energy barriers for CO_2_ absorption compared to Ag—Ag and Ag—Ni, therefore enhancing CO_2_ activation and catalytic efficiency for CO_2_ reduction. The strong interactions between carbon, nitrogen, and Ag atoms in the free‐standing Ag SAC contribute to its high CO selectivity, surpassing other silver configurations like Ag—Ag and Ag—Ni. Furthermore, the electronic structure and coordination environment confirm the superior performance of the Ag—N_3_ site, underscoring its effectiveness in CO_2_ electroreduction. These findings demonstrate the potential of Ag SACs to advance sustainable chemical synthesis and environmental remediation, offering a promising pathway for efficient carbon utilization.

## 4 Experimental Section

4.1

4.1.1

##### Materials

AgNO_3_ (≥99.99%), NH_4_OH (ACS reagent 28.0–30.0% NH_3_ basis), 2‐methylimidazole (99%), KHCO_3_ (ACS reagent, 99.7%), KOH (ACS reagent, ≥85%), H_2_O_2_ (ACS reagent, 30.0% in H_2_O), Nafion perfluorinated resin solution (5 wt% in mixture of lower aliphatic alcohols and water), and Ag nanopowder (<100 nm particle size, 99.5% trace metal basis) were procured from Sigma Aldrich. Ni foam (0.5 mm thickness, ≥ 95% porosity, >99.9 wt% purity) was obtained from MTI Corporation. ZnO nanoparticles (10–30 nm, >99%) were purchased from US Research Nanomaterials, Inc. Carbon paper gas diffusion layer and Fumasep‐FBM BPMs (soaked in 1.0 M NaCl for >24 h) were purchased from Fuel Cell Store. CO_2_ (>99.99%), N_2_ (>99.99%), and Ar (>99.99%) were acquired from Linde Gas & Equipment Inc. All chemicals were used in their original form, and solutions were prepared with double‐distilled water obtained from a Nanopure Diamond water purification system (18.2 MΩ cm).

##### Cell preparation

The two‐chamber reactor flow cell, constructed in‐house, features stainless steel anode and cathode housings (6 × 6 × 0.6 cm) with $\frac{1}{8}$″ NPT ports. A 4‐cm^2^ MEA was secured between a grade 2 titanium cathode and 316 stainless steel anode flow plates, each with serpentine channels that were 1.5 mm wide and deep, separated by 1 mm ribs. The MEA comprised a nickel foam anode, a BPM, and a free‐standing Ag SAC/Ni foam (2 × 2 cm^2^), which served as the GDE. The nickel foam at the anode functions as both the electrode and the catalyst for the oxygen evolution reaction under basic conditions. An Ag/AgCl (3.0 M KCl) electrode was used as the reference and connected to the cathode chamber. Polytetrafluoroethylene (PTFE) gaskets (1.5 mm thick) with 0.2 mm diameter holes for feed delivery provide a liquid and gas‐tight seal. The components were clamped between the stainless‐steel housings using six bolts (6.35 mm diameter, tightened to a torque of 3 N‐m).

##### Synthesis of Ag‐ZnO particles:

In a typical procedure, 1.55 g of ZnO nanoparticles and 0.4 g of AgNO_3_ were dispersed in 40 mL of water. A 0.50 mL of 30% NH_4_OH was mixed with the ZnO/AgNO_3_ solution, followed by the addition of 40 mL of 30% H_2_O_2_ dropwise, and stirred for 30 min. The formed Ag–ZnO nanoparticles were centrifuged, washed with water and dried at 60 °C overnight.

##### Fabrication of free‐standing single‐atom silver (Ag‐N‐C) electrode (1 atomic% Ag loading)

A 1.0 mL of 40 mg/mL of Ag–ZnO with 0.5% Nafion was cast on a 4 cm^2^ Ni foam and dried at 60°C. Subsequently, 1.0 mL of 400 mg mL^−1^ of 2‐methylimidazole (2mlm) with 0.5% Nafion was cast on the Ag–ZnO/Ni foam and dried at 60 °C. The formed 2‐mlm–Ag–ZnO/Ni foam was placed in a tube furnace and heated to 300 °C with a ramping rate of 5 °C min^−1^ under the stream of Ar gas. The temperature was maintained at 300 °C for 30 min, then gradually increased to 900 °C at a heating rate of 30 °C/min, followed by a 2 h hold at 900 °C to yield the free‐standing Ag–N–C electrode.

##### Material characterization

The morphology of the Ag SAC/Ni foam, as initially prepared, was examined using a SEM (Hitachi S‐5500) at 10 kV. High‐resolution transmission electron microscopy, atomic‐resolution HAADF‐STEM, and energy‐dispersive X‐ray spectroscopy analysis were performed using an FEI Titan 80–300 operated at 300 keV. For TEM analysis, samples were prepared by drop‐casting a sample/ethanol solution onto carbon film‐coated copper grids. XPS measurements were conducted using a Thermo Scientific XPS system. The samples were set at a take‐off angle of 90° (relative to the surface), with an X‐ray spot size of 400 μm with an Al K*α* monochromatic source. Binding energy values were calibrated against the C 1s signal at 284.5 eV. Data processing was performed using XPSPEAK 4.1 software. XRD patterns were acquired using a Phillips PW‐3710 utilizing Cu Ka1 radiation (1.5406 Å) to identify the crystalline phases of the synthesized Ag/ZnO and Ag/ZnO‐ZIF. Measurements spanned a 2*θ* range from 5° to 60° with a step size of 0.01°. Analysis of the XRD data was conducted using X’Pert High Score Plus software.

##### X‐ray absorption spectroscopy (*XAS) analysis*

The XAS analysis was performed using the as‐prepared catalyst samples in powder form. The samples were ultrasonicated in ethanol to ensure particle dispersion. They were then drop‐cast onto an appropriate sample holder and air dried. The samples contain more than 1 atomic% SAC and were stable in air. XAS measurements, including XANES and extended EXAFS at Ag K‐edge, were collected in total‐fluorescence‐yield mode using a 32‐element Ge detector under ambient conditions on the 06ID‐1 Hard X‐ray MicroAnalysis (HXMA) beamline at the Canadian Light Source (CLS). During the data collection, the CLS storage ring (2.9 GeV) was operated under 220 mA mode, and the HXMA superconducting wiggler was run at 1.9 T. The scan range was kept in an energy range of 25 314–26 260 eV for Ag K‐edge. Data collection configuration was using metal Ag foils as energy calibrations by in‐step calibration for every data set. The baseline of pre‐edge was subtracted, and the post‐edge was normalized in the spectra. EXAFS analysis was conducted using a Fourier transform on k^3^‐weighted EXAFS oscillations to evaluate the contribution of each bond pair to the Fourier transform peak. The coordination number fitting was performed in the R space with a K‐weight of 2 using IFEFFIT.^[^ ^72^ ^]^ The amplitude reduction factor S_0_^2^, the internal atomic distances R, the Debye–Waller factor σ^2^, and the edge‐energy shift ΔE were allowed to run freely.

##### CO_*2*_ *capture and electrochemical reduction process*

The CO_2_ capture and electrochemical conversion were performed in a single and continuous process as shown in Figure S1, Supporting Information. Before conducting electrochemical measurements, 400 mL of a 2.0 M KHCO_3_ solution was placed in a round‐bottom flask to capture CO_2_ in the catholyte. The flow rates of N_2_ and CO_2_ were adjusted to 180 sccm and 20 sccm, respectively, using a mass flow controller indicator located at the catholyte inlet of the round‐bottom flask. The catholyte was continuously circulated at a rate of 100 mL min^−1^ during all experiments through a peristaltic pump. Once the pH of the KHCO_3_ catholyte stabilized at 7.80, electrolysis was initiated using an electrochemical cell. All electrochemical tests were carried out in the two‐chamber flow cell reactor setup mentioned in the Cell preparation section. The anode compartment received 1.0 M KOH at a flow rate of 100 mL/min. A Solartron workstation was employed to perform LSV, CA, and CP in a three‐electrode cell system. The Ag/AgCl (3.0 M KCl) reference electrode was used, and the measured potentials were converted to the RHE scale (E vs. RHE) for comparison using the following equation:^[^ ^73^, ^74^ ^]^

(1) E (vs. RHE) = E (vs. Ag/AgCl) + 0.0591 V × pH + 0.210 V

Charge‐transfer resistance for CO_2_ reduction was assessed using EIS within a frequency range from 100 kHz to 10 mHz and an alternating current voltage amplitude of 10 mV. The EIS data were processed using Z‐View software to generate an equivalent electrical circuit model. Additionally, the reaction kinetics for CO2 reduction across various electrodes were evaluated through Tafel plots, derived from LSV curves recorded at a scan rate of 2 mV s^−1^ in a CO_2_‐saturated 2.0 M KHCO_3_ electrolyte.

The catholyte solution was continuously recycled to a reservoir purged with N_2_ gas at a flow rate of 180 sccm, facilitating the transport of reactor‐produced gases to an Agilent 990 Micro GC for analysis. Before being directed into the GC, the electrolyzer outlet headspace was passed through a condenser to remove condensable vapors. This high carbon concentration setup, maintained at a pH of 7.8, achieved the highest FE_CO_ efficiency in our electrolyzer. The reactor operated under galvanostatic conditions at current densities of 50, 100, or 200 mA cm^−2^. Gaseous products were analyzed using GC after 120 min of electrolysis to ensure complete saturation of the KHCO_3_ electrolyte. Experiments were performed in triplicate using a fresh free‐standing Ag SAC for each run. The KHCO_3_ solution in the reservoir, kept at ambient conditions, was refreshed after each assessment. GC analysis revealed that CO and H_2_ were the only gaseous reduction products. FE_CO_ represented the fraction of the total charge passed through the electrochemical cell that was utilized for CO production, with the remainder attributed to H_2_ production. The FE for CO and H_2_ production was calculated based on the concentrations detected by GC, according to the following formula:^[^ ^55^ ^]^

(2) $$\text{FE}=\frac{2FPrv_{g}}{RTj}$$

where 2 is the quantity of electrons required to reduce CO_2_ to form either CO or H_2_. *F* is Faraday's constant (96 485 C mol^−1^), *P* represents the atmospheric pressure (1.01 × 10^5^ Pa), *ν*_*g*_ denotes the volume concentration of the gas product in the exhaust stream as determined by online GC, *r* is the gas flow rate exiting the cell, measured by a flow meter (m^3^ s^−1^), R represents the ideal gas constant (8.314 J mol^−1^ K^−1^), *T* is the reaction temperature (298.15 K), and *j* is the steady‐state current density applied at the electrode. The electrochemical experiments were carried out under ambient room conditions at a temperature of 20 ± 2 °C.

##### Computational Methodology

Density functional theory (DFT) calculations were performed using the Vienna ab initio simulation package, with employing the projector augmented wave method and the Perdew–Burke–Ernzerhof (PBE) functional for the exchange‐correlation energy.^[^ ^69^, ^75^, ^76^ ^]^ The plane wave energy cutoff was set to 500 eV, and Brillouin zone sampling was performed with a 4 × 4 × 1 Monkhorst‐Pack k‐point grid. The convergence criteria of energy and forces were set to 1 × 10^−5^ eV and 0.05 eV Å^−1^, respectively. Van der Waals interactions were incorporated using the DFT‐D3 method developed by Grimme and colleagues, which addresses the dispersion forces often underestimated by standard DFT functionals.^[^ ^77^, ^78^ ^]^ Calculations were performed for two configurations: N‐pyridinic sites with single and diatomic silver atoms, and a Ni(111) slab with dimensions of 3 × 3 × 5. The N‐pyridinic structure was modeled using a periodically repeated single‐layer graphene structure embedded with Ag—N configurations to optimize the Ag–C–N framework. The graphene unit cell measured 5 × 5, with a vacuum slab height of 17 Å to prevent interlayer interactions. For the Ni(111) slab, a vacuum height of 20 Å was employed. All atoms were fully relaxed to meet stringent force and energy convergence thresholds.^[^ ^79^, ^80^, ^81^ ^]^ The Gibbs free energy changes associated with the CO_2_ reduction to CO were computed using the computational hydrogen electrode (CHE) model.^[^ ^82^ ^]^ This approach incorporated zero‐point energy (ZPE), and entropy (TS) contributions for the adsorbates at each stage of the reaction. The resulting free energy diagram provided insight into the thermodynamic viability of the CO_2_ RR to form CO and COH (Table S3, S4, S5, Supporting Information). The free energy for each species is calculated as follows: G = E_DFT_ + ZPE + ∫C_v_dT − TΔS, where E_DFT_ is the DFT‐optimized total energy, ZPE represents zero‐point vibrational energy, ∫C_v_dT is the heat capacity, T is the temperature, and ΔS is the entropy. Temperature is considered to be 298.15 K for this reaction. The electrochemical reduction of CO_2_ RR on a Ag catalyst's surface primarily produces CO. This process can be described using the CHE approach, which allows for the alignment of theoretical electrochemical potentials with experimental measurements. The reaction steps and their corresponding free energy changes are as follows:

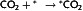




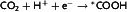




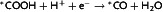




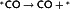

where * represents the clean adsorption site, *COOH denotes to the COOH molecule adsorbed at the active site, and *CO indicates the active site with CO adsorbed on it. Below are the free energy changes for each step:

Step 1: Adsorption of CO_2_


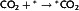




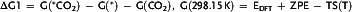




Step 2: *COOH formation

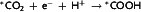




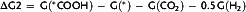




Step 3: *CO formation

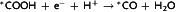




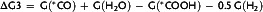




Step 4: CO desorption

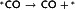




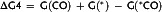




## Author Contributions

**M. Nur Hossain**: conceptualization (lead); data curation (lead); investigation (lead); methodology (lead); visualization (lead); writing—original draft (lead). **Ali Malek**: data curation (lead); visualization (supporting); writing—review and editing (supporting). **Zhangsen Chen**: data curation (supporting). **Lei Zhang**: conceptualization (lead); funding acquisition (lead); project administration (lead); supervision (lead); writing—review and editing (lead). **Shuhui Sun**: supervision (lead); funding acquisition (supporting). **Hanshuo Liu**: data curation (supporting). **Roberto Neagu**: conceptualization (supporting); investigation (supporting). **Jigang Zhou**: data curation (supporting). **Hui Yuan**: data curation (supporting). **Christopher S. Allen**: data curation (supporting). **Gianluigi Botton**: investigation (supporting).

## Conflict of Interest

The authors declare no conflict of interest.

## Supporting information

Supplementary Material

## Data Availability

The data that support the findings of this study are available from the corresponding author upon reasonable request.
