# Supplementary material for: Efficient Electrochemical CO2 Reduction Using AgN3 Single‐Atom Sites Embedded in Free‐Standing Electrodes for Flow Cell Applications
Source: Small Sci. 2025 Jul 6;5(8):2400643. doi: 10.1002/smsc.202400643 (PMC12362748; doi:10.1002/smsc.202400643)
Supplement: Supplementary file 1 — Supplementary Material [file SMSC-5-2400643-s001.pdf]

Supporting Information

Efficient Electrochemical CO_2 Reduction Using AgN_3 Single-Atom Sites Embedded in Free-Standing Electrodes for Flow Cell Applications

M. Nur Hossain[1] | Ali Malek[1] | Zhangsen Chen[2] | Lei Zhang[1,*] | Shuhui Sun[2,*] | Hanshuo Liu[1] | Roberto Neagu[1] | Jigang Zhou[3] | Hui Yuan[4] | Christopher S. Allen[5,6,*] | Gianluigi Botton[3,4,6]

1 Dr. M. Nur Hossain, Dr. Ali Malek, Dr. Lei Zhang, Dr. Hanshuo Liu, Dr. Roberto Neagu
Clean Energy Innovation Research Center
National Research Council of Canada
Vancouver, BC, V6T 1W5, Canada
E-mail: lei.zhang@nrc-cnrc.gc.ca

2 Dr. Zhangsen Chen, Prof. Shuhui Sun,
Centre Énergie Matériaux Télécommunications
Institut National de la Recherche Scientifique (INRS)
Varennes, Québec J3X 1P7, Canada
E-mail: shuhui.sun@inrs.ca

3 Dr. Jigang Zhou, Prof. Gianluigi Botton
Canadian Light Source
Saskatoon, Saskatchewan, S7N 2V3, Canada

4 Dr. Hui Yuan, Prof. Gianluigi Botton
Department of Materials Science and Engineering
McMaster University
Hamilton, Ontario, L8S 4L7, Canada

5 Dr. Christopher S. Allen
Department of Materials
University of Oxford
Parks Road, Oxford OX1 3PH, United Kingdom

6 Dr. Christopher S. Allen, Prof. Gianluigi Botton
electron Physical Science Imaging Center
Diamond Light Source Ltd., Harwell Science and Innovation Campus
Chilton, Didcot OX11 0DE, United Kingdom

Table of Contents

Figure S1: Schematic representation of the bicarbonate electrolyzer system detailing the flow of reactants and products..3
Figure S1: XRD spectra……….4
Figure S3: SEM image of the bare Ni foam...5
Figure S4: Photos of a flexible, free-standing Ag SAC electrode…………………………………………………………………………………………………6
Figure S5: XPS spectra of free-standing Ag SAC electrode...7
Figure S6: K-space spectra of Ag SAC electrode and foil..8
Figure S7: Nyquist plots and Tafel plots………………………………………………………………………………………………………..………….…9
Figure S8: LSV, CA and CP curves..10
Figure S9: HAADF-STEM imge, Elemental mapping and XPS spectra……………………………………………………………………………………..11
Figure S10: Binding energies of the intermediates on Ni and Ag-Ni sites………...12
Figure S11: Adsorption and desorption configurations on Ag-N_3 and Ag-Ag sites…....………..13
Figure S12: Binding energies of the intermediates on Ag-N_3 and Ag-Ag sites…....…………..14
Table S1: Values of the elements in an equivalent electric circuit fitted in the Nyquist plots…………………………………………………………..……………15
Table S2: Performance comparison of Ag-based electrocatalysts………………………………………………………………………………………….……15
Table S3: Gas phase molecules and thermodynamic quantities 16
Table S4: DFT calculations for single-atom site...16
Table S5: DFT calculations for dimer site………...16

SUPPORTING INFORMATION

Figure S1. Schematic representation of the bicarbonate electrolyzer system detailing the flow of reactants and products. A peristaltic pump circulates 1 M KOH at a flow rate of 100 mL min^{-1} from the anode flow plate to the anode, then recycles it back to the KOH reservoir while venting O_2. Simultaneously, another peristaltic pump delivers 2.0 M $KHCO_3$ at 100 mL min^{-1} to the cathode flow plate, recycling the resulting gas and liquid mixture back to the $KHCO_3$ reservoir. A mass flow controller regulates a constant N_2 flow of 160 sccm through the headspace of the 2.0 M $KHCO_3$ reservoir to transport CO, H_2, and CO_2 to the GC analyzer for measurement.

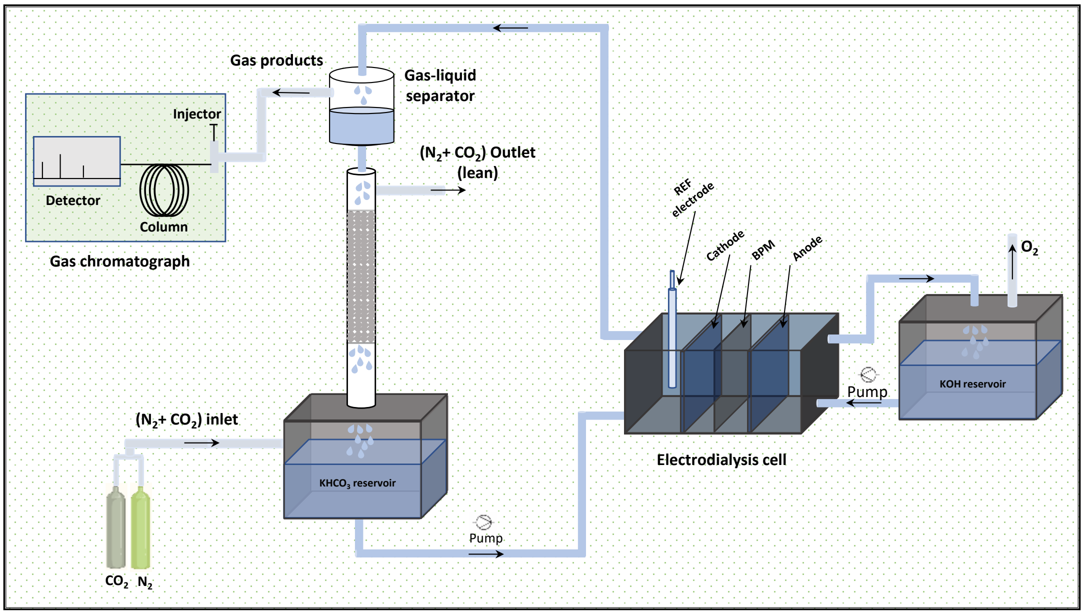

Figure S2. XRD spectra of the synthesized Ag/ZnO and Ag/ZnO-ZIF.

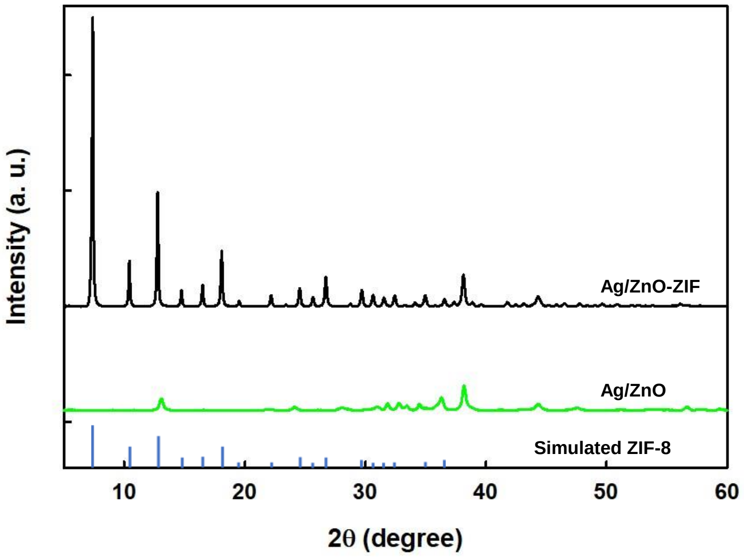

Figure S3. (A) SEM image of the bare Ni foam. (B) High-magnification SEM image of the bare Ni foam.

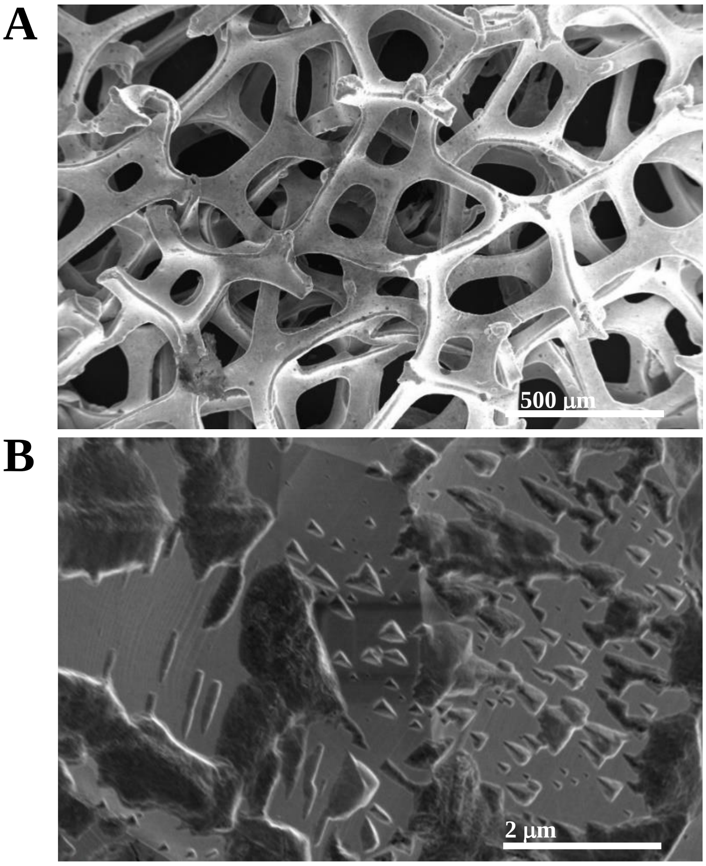

Figure S4. Photos of a flexible, free-standing Ag SAC electrode. (A-D) Before electrolysis. (E) After a stability test at 100 mA cm^{-2} for 20 hours.

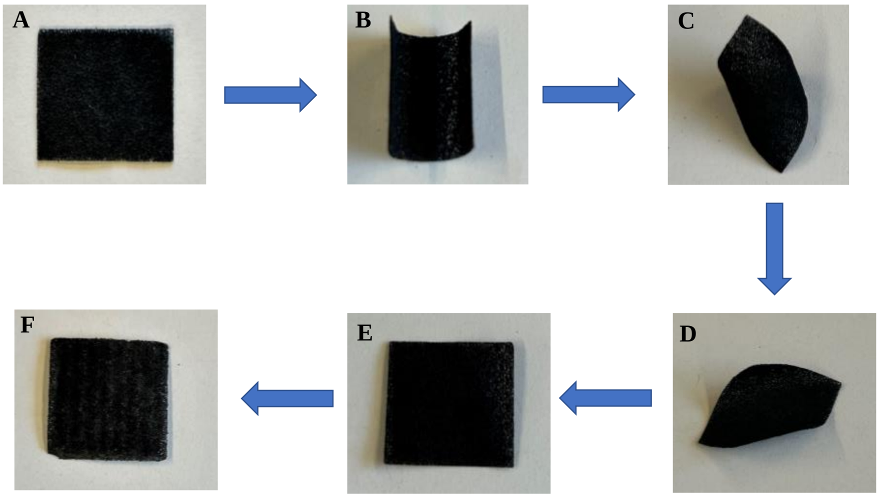

Figure S5. The XPS survey spectra of the free-standing Ag SAC electrode.

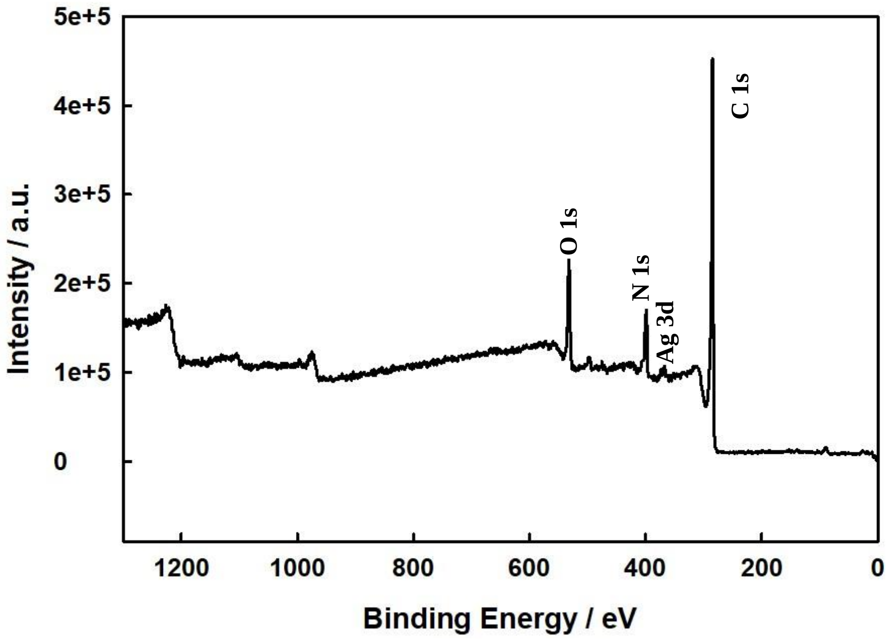

Figure S6. K-space spectra of the free-standing Ag SAC electrode and Ag foil.

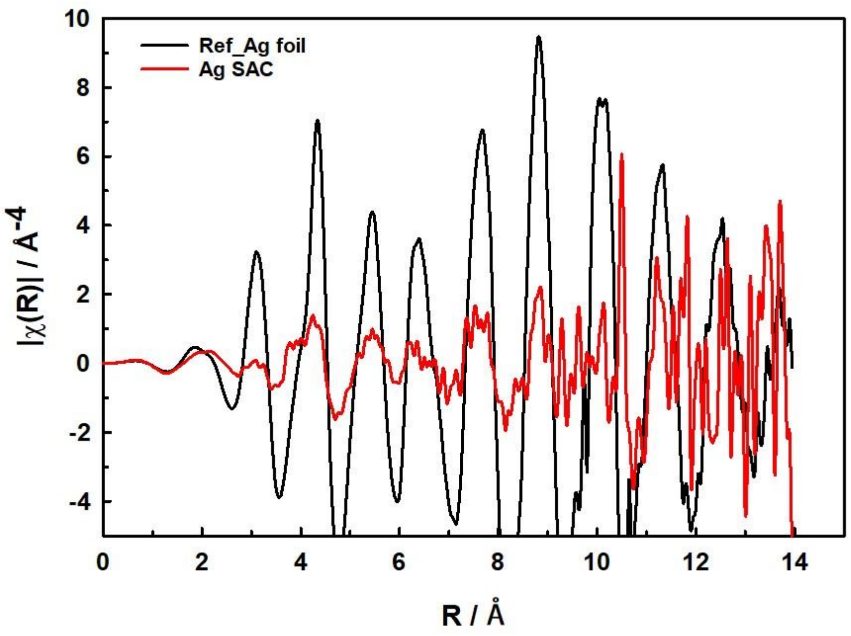

Figure S7. (A) Nyquist plots for various electrodes at -0.35 V (vs. RHE) in a CO_2-saturated 2.0 M $KHCO_3$ solution. (B) Nyquist plots at different potentials for the Ag SAC electrode; inset: the equivalent electrical circuit used to fit the experimental EIS data, where Rs is the solution resistance, Rct is the charge-transfer resistance, and CPE is the constant phase element. (C) Tafel plots for the electrochemical reduction of CO_2 at different electrodes.

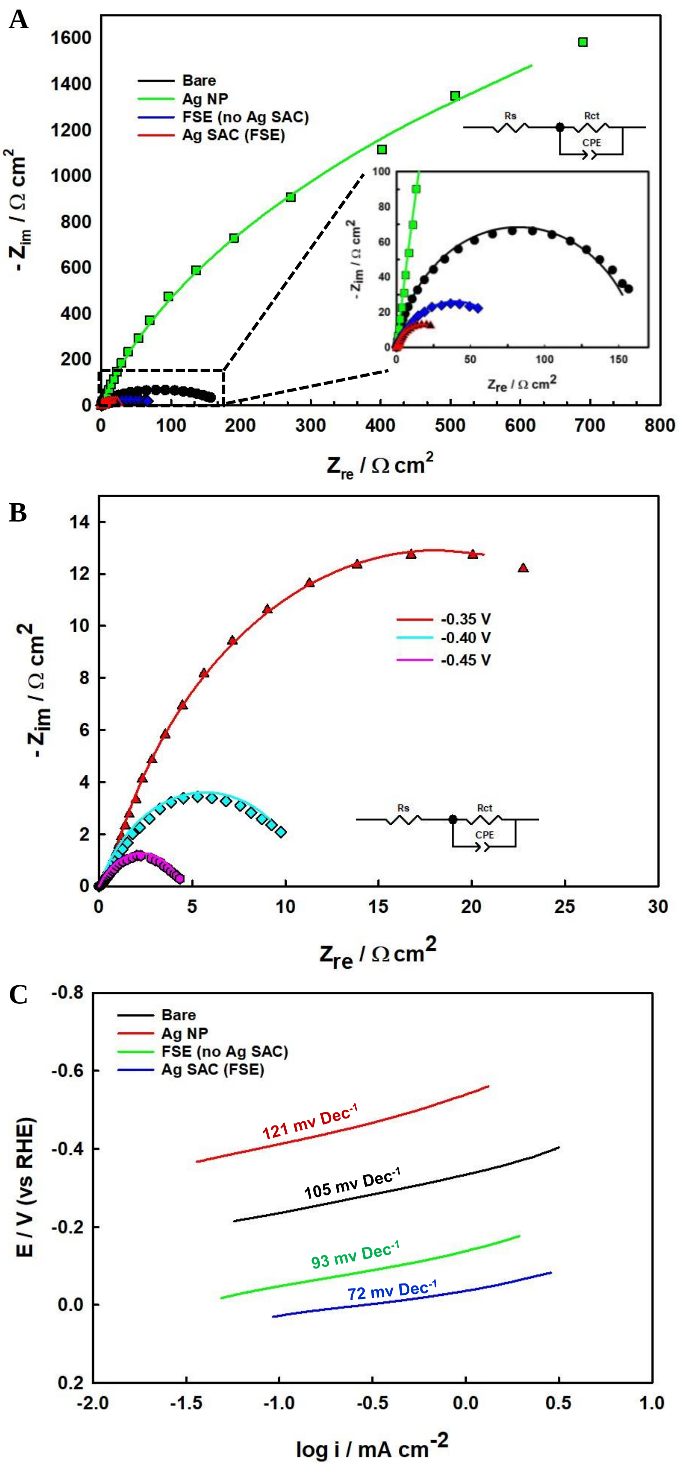

Figure S8. (A) LSV curves obtained at a scan rate of 20 mV s^{-1}, (B) CA curves recorded at -0.35 V (vs RHE), and (C) CP curves measured at -100 mA cm^{-2}. Broken lines represent Ar-saturated electrolytes and solid lines represent CO_2-saturated electrolytes.

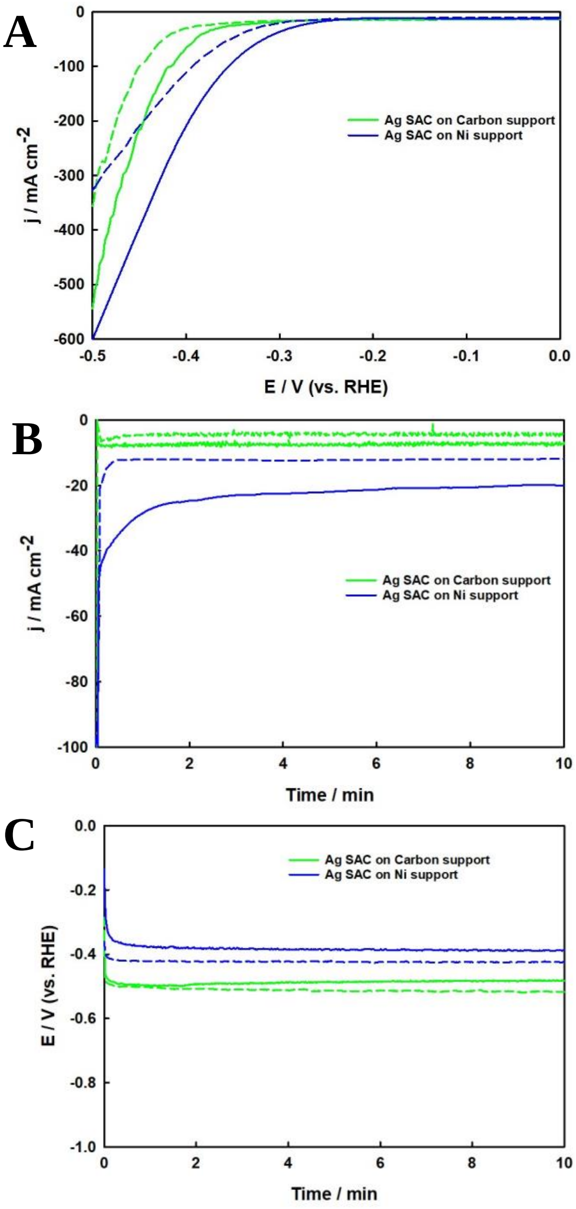

Figure S9. Post-electrolysis characterization of the free-standing Ag SAC electrode after 20 hours of electrochemical CO_2 reduction at −100 mA cm^{-2}: (A) Aberration-corrected HAADF-STEM image. (B) Elemental mapping showing the distribution of silver (Ag) in green, carbon (C) in cyan, and nitrogen (N) in yellow. (C) XPS survey spectra. (D) High-resolution XPS spectra of Ag 3d.

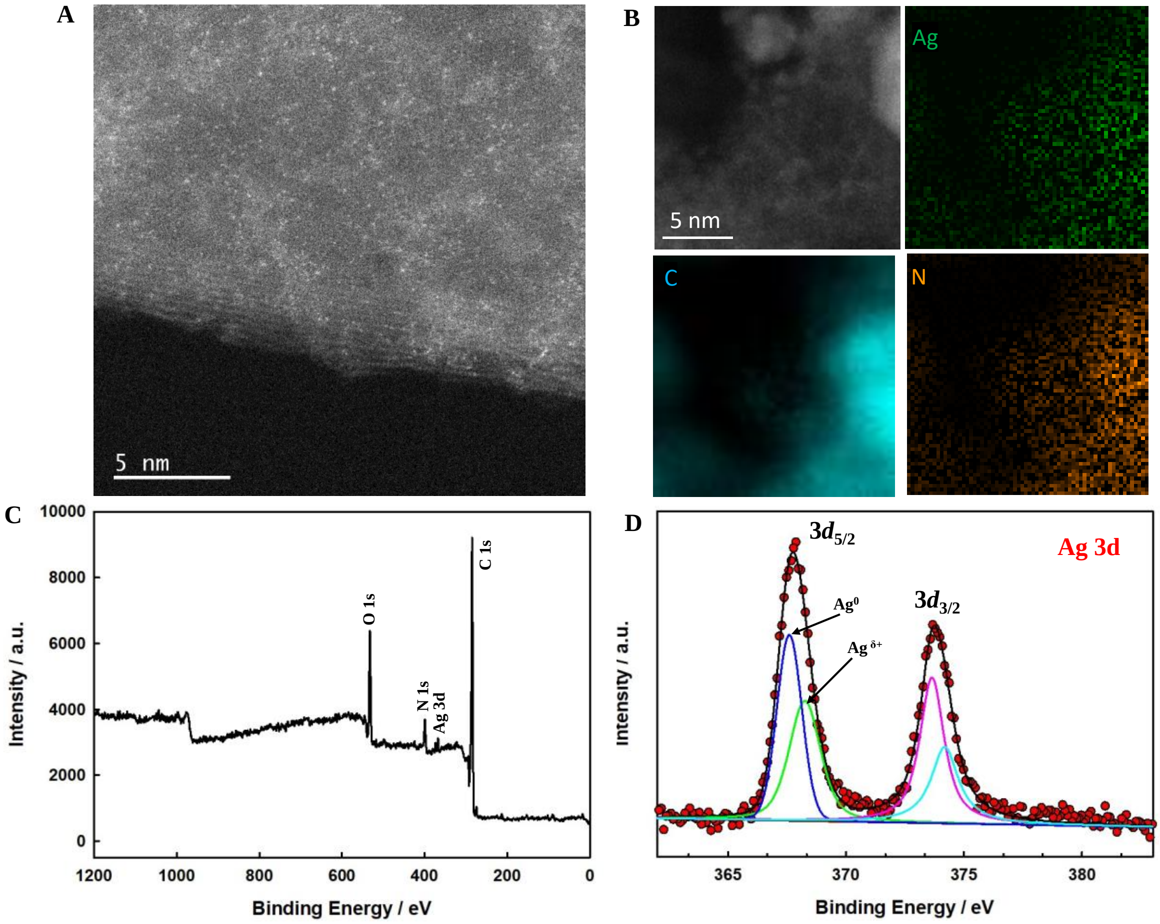

SUPPORTING INFORMATION

Figure S10: Binding energies of the intermediates on Ni and Ag-Ni for the pathways of (A) CO and (B) H_2.

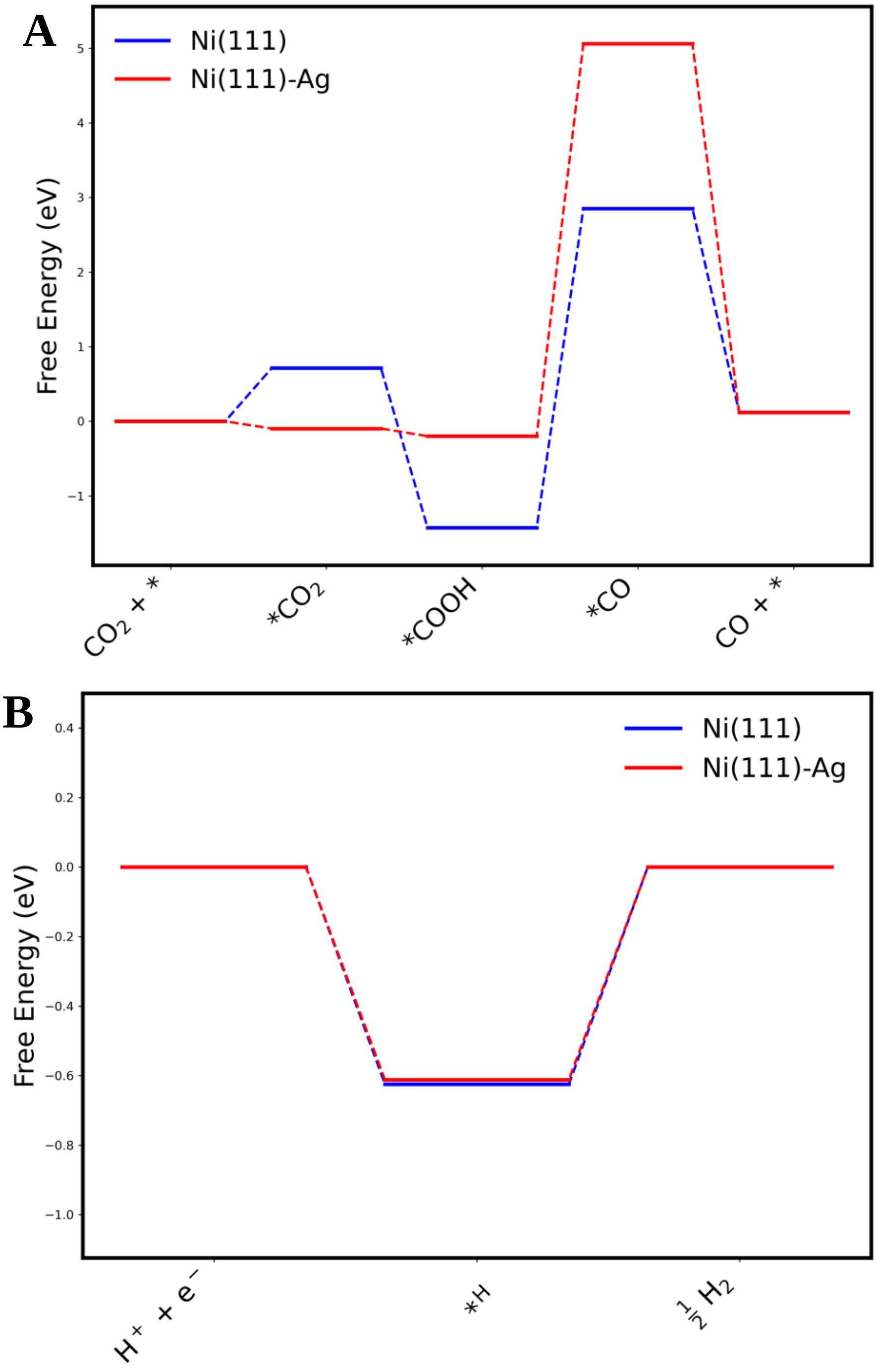

Figure S11: *COO, *COOH and *CO adsorption and CO desorption configurations on (A) Ag-N_3 and (B) Ag-Ag. The green dashed lines between Ag and species and slab models represent the weak physical adsorptions. Color code: C, black; N, blue; Ag, silver; O, red; H, light red.

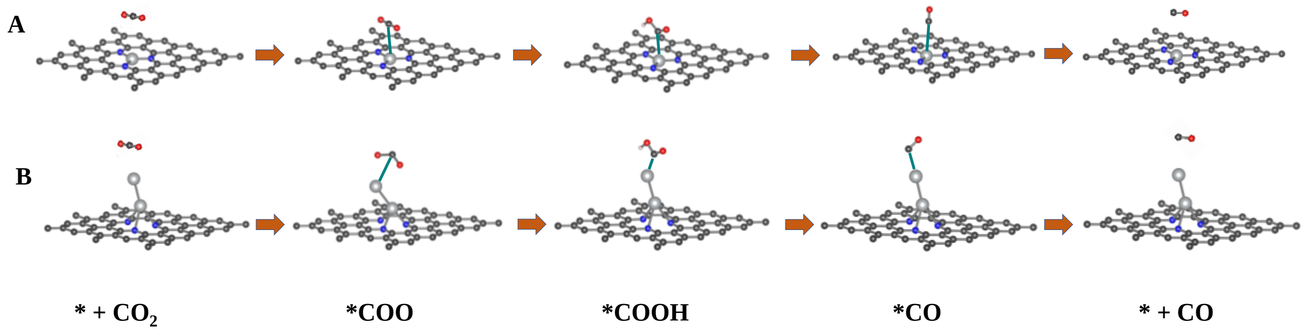

Figure S12: Binding energies of the intermediates on Ag-N_3 and Ag-Ag along hydrocarbon production pathways.

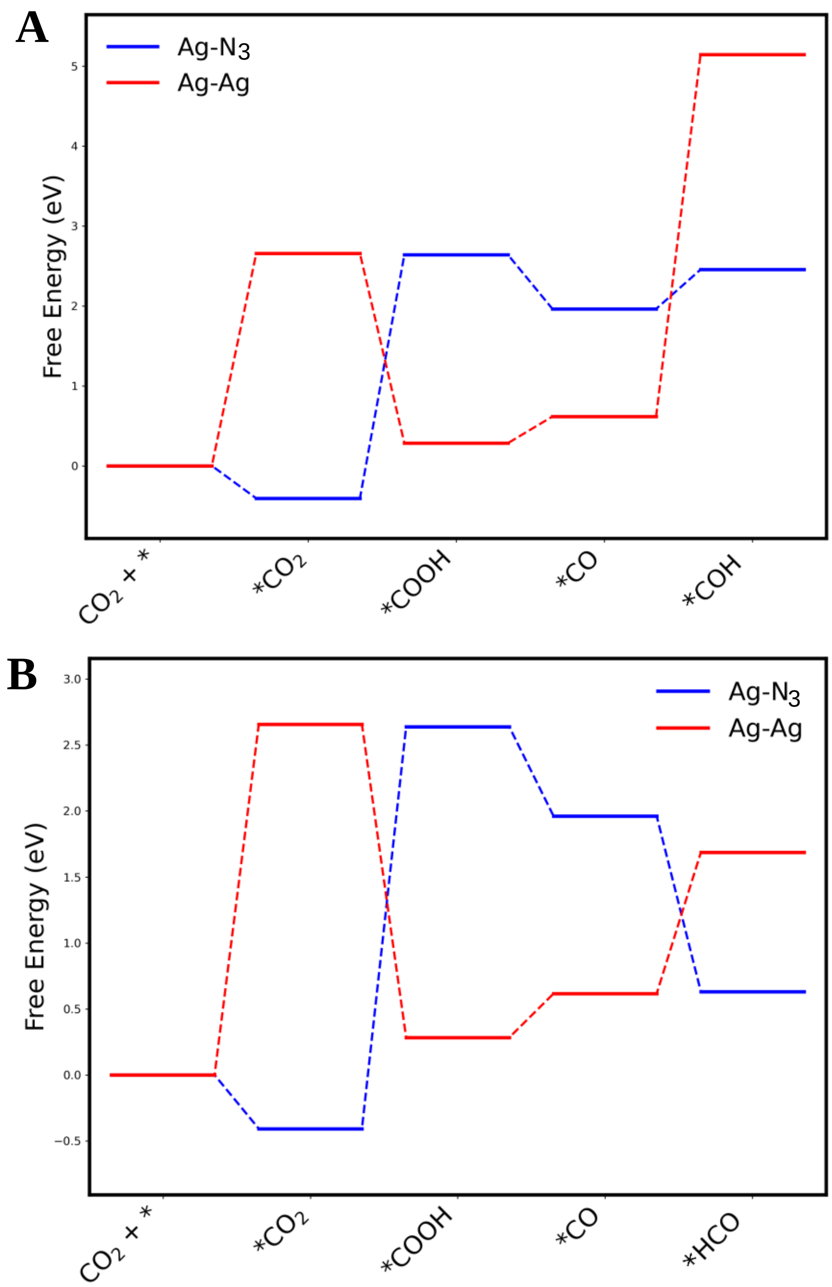

Table S1. Values of the elements in an equivalent electric circuit fitted in the Nyquist plots shown in Fig. S8A and S8B (error percentage for each element is provided in parentheses)

Elements	Bare (%)	Ag NP (%)	FSE (no Ag SAC) (%)	Ag SAC (FSE) (%)		
				-0.35 V	-0.40 V	-0.45 V
Rs (Ω cm^2)	1.69 (0.49)	31.15 (0.66)	4.19 (2.98)	1.91 (1.96)	1.66 (1.39)	0.92 (1.17)
CPE-T (μF cm^2)	3.55 (1.30)	2.52 (1.06)	60.56 (1.51)	209.75 (2.24)	241.60 (2.97)	306.31 (1.99)
CPE-P	0.82 (0.23)	0.91 (0.36)	0.77 (2.39)	0.80 (1.45)	0.73 (1.60)	0.72 (1.73)
Rct (Ω cm^2)	149.40 (0.75)	678 (3.25)	55.41 (2.24)	24.72 (1.94)	9.21 (1.53)	4.12 (2.29)

Rs: solution resistance; Rct: charge transfer resistance; CPE-T/CPE-P: constant phase element

Table S2. Performance comparison of various reported Ag-based electrocatalysts in CO_2 reduction reactions.

Catalyst	Electrolyte	Onset potential (V)	Potential; Current density	FE_{CO} (%)	Stability	Ref.
Ag_1-G	0.5 M $KHCO_3$	-0.4	- 0.7 V; 6.47 mA cm^{-2}	79.2	36 h (11.8 mA cm^{-2})	[1]
Ag_2-G	0.5 M $KHCO_3$	-0.25	-0.7; 11.87 mA cm^{-2}	93.4		
Ag−N_3/PCNC	0.1 M $KHCO_3$	-0.24	-0.37 V; 2.4 mA cm^{-2} (Jco)	94.6	40 h (2.4 mA cm^{-2})	[2]
Pre-oxidized Ag	0.1 M $KHCO_3$	-0.5	-0.6 V	90	3 h (~2.5 mA cm^{-2})	[3]
Tri-Ag-NPs	0.1 M $KHCO_3$	-0.206	-0.856 V	96.8	14 h (1.2 mA cm^{-2})	[4]
Oxide derived Ag	0.1 M $KHCO_3$	-0.4	-0.6 V	80	2 h (~0.35 mA cm^{-2})	[5]
Ag_1/MnO_2	0.5 M $KHCO_3$	-	-0.85 V; 2.0 mA cm^{-2} (Jco)	95.7	9 h (~5.0 mA cm^{-2})	[6]
Ag/CoO/$NCNS_1$	0.5 M $KHCO_3$	-0.24	-0.5 V	88	7 h (~15.0 mA cm^{-2})	[7]
p-Ag/CP	0.1 M$KHCO_3$	~-0.6	-1.0 V; ~10.0 mA cm^{-2}	96.7	27 (~7.0 mA cm^{-2})	[8]
Ag/g-C_3N_4	1.0 M$KHCO_3$	-0.19	-0.7 V; ~15.0 mA cm^{-2}	94	20 (11.5 mA cm^{-2})	[9]
d Ag	0.5 M $KHCO_3$	-	-0.83 V; 2.5 mA cm^{-2} (Jco)	62	1.5 h (2.5 mA cm^{-2})	[10]
CP-Ag-1.3	0.1 M $KHCO_3$	-0.3	-1.1 V; ~17.5 mA cm^{-2}	90	24 h (~6.5 mA cm^{-2})	[11]
Ag/CNT-C OOH	0.1 M $KHCO_3$	~-0.6	-1.3V; 1.5 mA cm^{-2} (Jco)	80	10 h (~15.0 mA cm^{-2})	[12]
Nanoporous Ag	0.5 M $KHCO_3$	-	−0.41 V; 3.0 mA cm^{-2} (Jco)	65	8 h (~10.0 mA cm^{-2})	[13]
**Ag-N_3**	**2.0 M $KHCO_3$**	**-0.27**	**-0.4V; 100.0 mA cm^{-2} (60.2 mA cm^{-2} Jco)**	**60.2**	**20 h (100 mA cm^{-2})**	**This work**

Table S3: Gas phase molecules and their thermodynamic quantities (eV)

Molecule	EDFT (eV)	ZPE (eV)	∫CvdT (eV)	TS (eV)	G (eV)
H_2O	-14.21	0.56	0.1	0.67	-14.22
CO_2	-22.95	0.31	0.1	0.66	-22.79
H_2	-6.75925518	0.27	0.09	0.43	-6.94
CO	-14.78	0.13	0.09	0.67	-15.22
HCOOH	-29.87	0.89	0.11	1.05	-29.71

Table S4: DFT calculations for single-atom site

System	Energy (eV)	ZPE (eV)	TS (eV)	G (eV)
GN-Ag	-440.1621113	0.0	0.0	-440.1621113
GN-Ag-CO_2	-463.3186545	0.0392	0.4896	-463.7690545
GN3-Ag-COOH	-464.9337893	0.612945	0.224709	-464.5455533
GN3-Ag-CO	-455.0568717	0.147962	0.283352	-455.1922617
GN3-Ag-H	-441.5233397	0.167515	0.016964	-441.3727887

Table S5: DFT calculations for dimer site

System	Energy (eV)	ZPE (eV)	TS (eV)	G (eV)
GN-Ag_2	-451.10788675	0.0	0.0	-451.10788675
GN-Ag_2-CO_2	-470.93415232	0.0388	0.7563	-471.65165232
GN3-Ag_2-COOH	-474.05866424	0.0755	0.7989	-474.78206424
GN3-Ag_2-CO	-466.38664652	0.0197	0.4079	-466.77484652
GN3-Ag_2-H	-451.17082898	0.0197	0.0663	-451.21742898

References

[1] Y. Li, C. Chen, R. Cao, Z. Pan, H. He, K. Zhou, Appl Catal B 2020, 268, 118747.
[2] R. Sui, J. Pei, J. Fang, X. Zhang, Y. Zhang, F. Wei, W. Chen, Z. Hu, S. Hu, W. Zhu, Z. Zhuang. ACS Appl. Mater. Interfaces 2021, 13, 17736−17744.
[3] H. Mistry, Y. W. Choi, A. Bagger, F. Scholten, C. S. Bonifacio, I. Sinev, N. J. Divins, I. Zegkinolou, H. S. Jeon, E. A. Stach, J. C. Yang, J. Rossmeisl, B. R. Cuenya, Angew. Chem. Int. Ed. 2017, 56, 11394-11398.
[4] S. Liu, H. Tao, L. Zeng, Q. Liu, Z. Xu, Q. Liu, J. L. Luo, J. Am. Chem. Soc. 2017, 139, 2160-2163.
[5] M. Ma, B. J. Trześniewski, J. Xie, W. A. Smith, Angew. Chem. Int. Ed. 2016, 55, 9748-9752.
[6] N. Zhang, X. Zhang, L. Tao, P. Jiang, C. Ye, R. Lin, Z. Huang, A. Li, D. Pang, H. Yan, Y. Wang, P. Xu, S. An, Q. Zhang, L. Liu, S. Du, X. Han, D. Wang, Y. Li, Angew. Chem. Int. Ed. 2020, 60, 6170-6176.
[7] M. Tariq, F. Nasim, R. Mansha, S. Gul, W. A. Shah, M. A. Nadeem, Energy Fuels 2025, 39, 2665−2674.
[8] J. Ding, T. Wei, T. Hou, W. Liu, Q. Liu, H. Zhang, J. Luo, X. Liu, Nanoscale 2024, 16, 10628− 10636.
[9] S. Zhang, Z. Mo, J. wang, H. Liu, P. Liu, D. Hu, T. Tan, C. Wang, Electrochim. Acta 2021, 390, 138831.
[10] Y. S. Ham, S. Choe, M. J. Kim, T. Lim, S.-K. Kim, J. J. Kim, Appl. Catal. BEnviron. 2017, 208, 35-43
[11] W. Guo, K. Shim, F. O. Odongo Ngome, Y. H. Moon, S.-Y. Choi, Y.-T. Kim, Journal of CO_2 Utilization 2020, 41, 101242.
[12] J. Sun, J. Xu, H. Jiang, X. Zhang, D. Niu, ChemElectroChem 2020, 7 (8), 1869-1876.
[13] Q. Lu, J. Rosen, Y. Zhou, G. S. Hutchings, Y. C. Kimmel, J. G. Chen, F. Jiao, Nat. Commun. 2014, 5, 3242.
